# Microsatellite markers of water buffalo, *Bubalus bubalis *- development, characterisation and linkage disequilibrium studies

**DOI:** 10.1186/1471-2156-10-68

**Published:** 2009-10-21

**Authors:** Muniyandi Nagarajan, Niraj Kumar, Gopala Nishanth, Ramachandran Haribaskar, Karthikeyani Paranthaman, Jalaj Gupta, Manish Mishra, R Vaidhegi, Shantanu Kumar, Amresh K Ranjan, Satish Kumar

**Affiliations:** 1 Centre for Cellular and Molecular Biology, Uppal Road, Hyderabad-500007, India

## Abstract

**Background:**

Microsatellite markers are highly polymorphic and widely used in genome mapping and population genetic studies in livestock species. River buffalo, *Bubalus bubalis *is an economically important livestock species, though only a limited number of microsatellite markers have been reported thus far in this species.

**Results:**

In the present study, using two different approaches 571 microsatellite markers have been characterized for water buffalo. Of the 571 microsatellite markers, 498 were polymorphic with average heterozygosity of 0.51 on a panel of 24 unrelated buffalo. Fisher exact test was used to detect LD between the marker pairs. Among the 137550 pairs of marker combination, 14.58% pairs showed significant LD (P < 0.05). Further to check the suitability of these microsatellite markers to map these on a radiation hybrid map of buffalo genome, the markers were tested on Chinese hamster genomic DNA for amplification. Only seven of these markers showed amplification in Chinese hamster, and thus 564, of these can be added to the radiation hybrid map of this species.

**Conclusion:**

The high conservation of cattle microsatellite loci in water buffalo promises the usefulness of the cattle microsatellites markers on buffalo. The polymorphic markers characterised in this study will contribute to genetic linkage and radiation hybrid mapping of water buffalo and population genetic studies.

## Background

Genetic maps provide new insights into genome structure and chromosomal architecture of the genome, and also serve as framework for identification and location of genes linked with economically important traits. Except for water buffalo, the genetic maps have been reported for most of the important livestock species. Water buffalo (*Bubalus bubalis*) is one among the important livestock species and has a wide geographical distribution in the Indian sub-continent, Middle East, Eastern Europe and several other Asian countries. To develop genetic maps of water buffalo, identification and characterisation of polymorphic microsatellite markers is a prerequisite.

Microsatellite markers are tandemly repeated short DNA sequences, often highly polymorphic. These have proved useful in marker assisted selection of desirable traits to which they are linked; hence are the markers of choice for genome mapping studies [1]. The repeat-flanking sequences of microsatellite loci are often conserved between closely related species [2,3], thus allowing cross species amplification on related species for which microsatellite markers have not been developed. Such an approach has been proved effective by several previous studies including by us [4]. A large number of microsatellite loci have been characterized for domesticated cattle [5-7], although in the recent past a very few studies have used cattle microsatellite markers to amplify on water buffalo genome [2,4]. The number of microsatellite markers developed for buffalo has been very small. In our earlier study, we have used 108 cattle markers to amplify buffalo microsatellite loci, of the 108 markers 81 were amplified and 61 were polymorphic in buffalo genome. Most importantly, no de-novo microsatellite markers have been reported for this species. In the present study, we have characterised 571 microsatellite markers for water buffalo using two different approaches and tested their suitability in construction of genome map for water buffalo.

## Results

**Figure 1:**
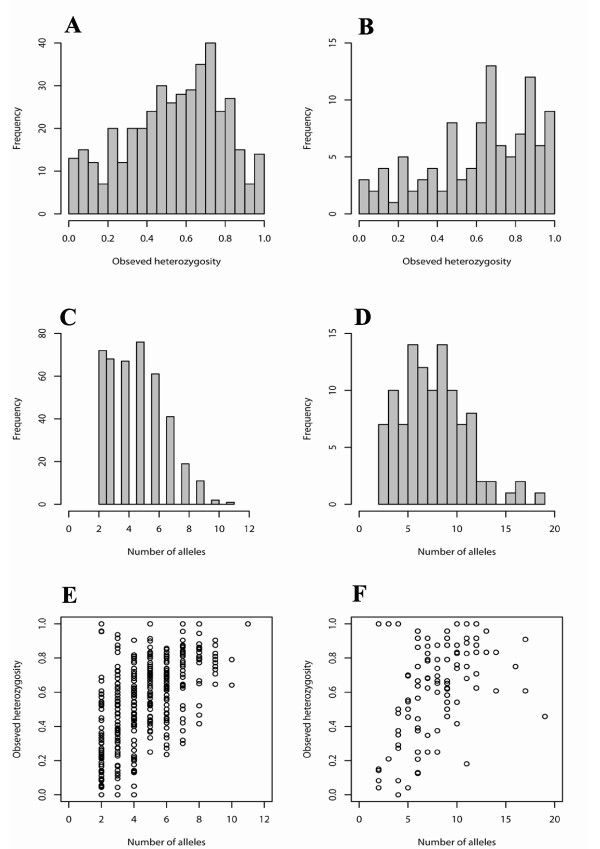
**Characteristics of *Bubalus bubalis *polymorphic microsatellite markers**. A & C, heterozygosity and number of alleles observed for cattle microsatellite markers on buffalo genome. B & D, observed heterozygosity and number of alleles of microsatellite markers isolated through genomic library construction of buffalo genome. E & F, the relationship between the number of alleles and the heterozygosity of cattle markers and buffalo markers.

A total of five hundred and ninety four cattle microsatellite primer pairs were tested for amplification on water buffalo genomic DNA. Of the 594 primer pairs tested, 457 (76.9%) gave discrete amplification products. Of these amplified products, 391 (85.5%) were polymorphic [see Additional file 1] and the remaining 66 (14.5%) were monomorphic [see Additional file 2] on a panel of 24 unrelated Murrah buffaloes. The average number of alleles per polymorphic locus was 4.64, ranging from 2 to11 (Figure 1C & Additional file 1). The values of observed heterozygosity ranged from 0 to 1 (52.08%) against the expected heterozygosity values from 0.04 to 0.88 (Figure 1A & Additional file 1). Of the 391 polymorphic loci, 24 loci showed significant departure from Hardy-Weinberg equilibrium after applying bonferroni correction.

To further generate new microsatellite markers for buffalo genome, we constructed a small insert library. Four hundred sixty clones hybridizing to CA/GA repeats were picked up and the plasmid DNA was isolated and inserts were sequenced from both directions. Out of these, 303 sequences contained microsatellite repeat motif, from which 177 sequences were selected to design primers based on the GC content and length of the flanking region. Of which, 114 primer pairs amplified discrete products [see Additional file 3 &4] and 107 of the corresponding loci revealed extensive polymorphism. The number of alleles per locus ranged from 2 to 19 with an average of 8.04 (Figure 1D & Additional file 3). The observed heterozygosity of the polymorphic markers ranged from 0 to 1 (62.29%) against the expected heterozygosity values from 0.05 to 0.93 (Figure 1B & Additional file 3). Of the 107 polymorphic loci, 32 loci significantly (P < 0.05) deviated from the Hardy-Weinberg equilibrium after the bonferroni correction [see Additional file 3].

Thus, we have characterised 498 polymorphic microsatellite markers in this study for an economically important livestock species, *Bubalus bubalis*. The average number of alleles observed was 5.37 for all the 498 markers, whereas the average heterozygosity was 0.51. The markers with a high number of alleles tended to be more heterozygous whereas the markers with a small number of alleles exhibited diverse level of heterozygosity (Figure 1E &1F).

### Linkage disequilibrium

**Figure 2:**
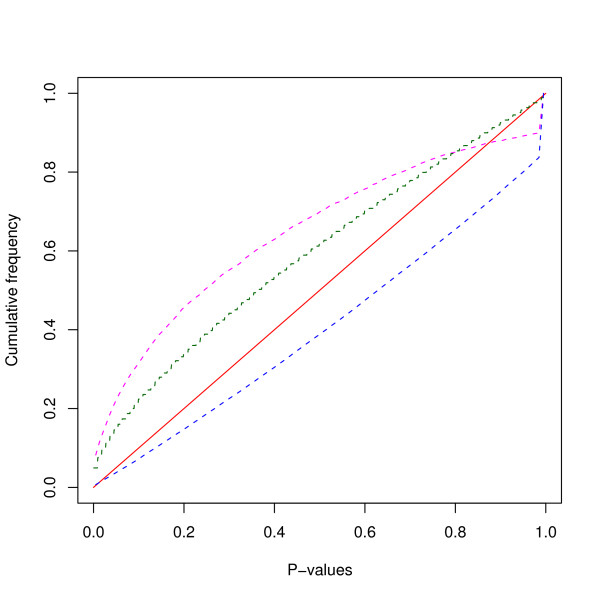
**Cumulative frequency distribution plot**. The cumulative frequency distribution of LD P-value of cattle markers (blue line), buffalo markers (pink line) all the marker (green line). The red line represents the expected distribution under random allelic segregation.

**Figure 3:**
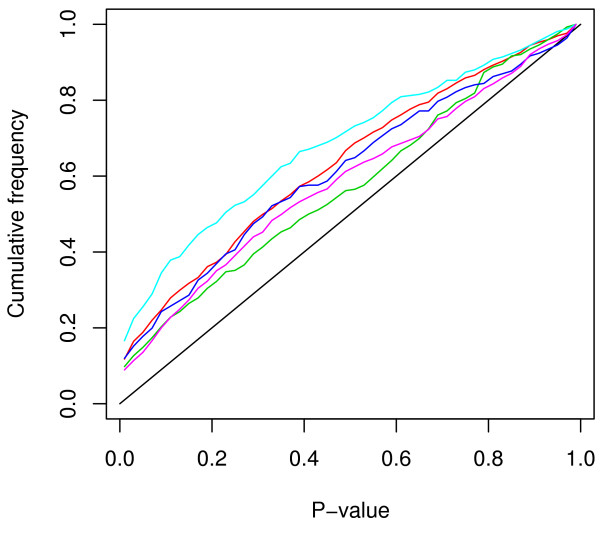
**Cumulative frequency distribution plot**. The cumulative frequency distribution of LD P-value between cattle intra chromosomal markers on buffalo genome: BTA1 (red line), BTA9 (light green), BTA10 (blue line), BTA11 (light blue line), BTA14 (magenta line). The black line represents the expected distribution under random allelic segregation.

The statistical significance of the linkage disequilibrium among 525 microsatellite loci, including 27 markers characterised by us earlier [4], was tested by Fisher's exact test. LD P-values were obtained for 137550 pairs of markers combinations. Out of these, 20064 (14.58%) pairs showed significant LD at P < 0.05 and 10380 (7.5%) pairs at P < 0.01. Since large number of tests were performed for several markers combinations it was expected that some pairs of markers would show significant LD. Thus, a Bonferroni correction for multiple testing would result in loss of power to detect LD [8,9]. Instead, we made a plot for the cumulative frequency distribution of P-values. Under the null hypothesis of random allelic association, the expected cumulative frequency distribution of P-values is on the diagonal of the graph in Figure 2. The cumulative frequency of P values significantly departed from the distribution expected under the random allelic association (Figure 2) suggesting substantial level of LD between the markers used in this study. We also checked the LD significance for cattle and buffalo markers independently. Out of 87153 pairs of cattle markers, 12.9% showed significant LD (P < 0.05) whereas 47.2% pairs of buffalo markers (out of 5671 pairs) showed significant LD (P < 0.05). Further, we wanted to know how the LD behaved between intra chromosomal markers. Thus we sorted out the cattle markers, based on the cattle map and we chose five chromosomes (BTA1, BTA9, BTA10, BTA11 & BTA14) with reasonable number of markers (24 to 35). As expected the distribution of the P values between syntenic markers largely departed from the diagonal (Figure 3), indicating strong LD for syntenic markers in all the five tested chromosomes. The number of pairs showing significant LD (P < 0.05) varies from 12.6% in BTA14 to 23.7% in BTA11.

## Discussion

The usefulness of cattle microsatellite markers in molecular genetic studies have been reported for several bovidae species and showed extensive genomic conservation between cattle and other bovidae species. However, the extent of conservation is varied between species. The percentage of conservation of cattle microsatellite loci in water buffalo obtained (76.9%) in the present study was comparable with other bovidae species [10-13] and supports the previous finding on the relative usefulness of cattle primers across closely related bovidae species. Among the amplified cattle markers, 85% of the markers were polymorphic, this number was slightly high when compared with previous studies on water buffalo [2,4]. In the present study, the average heterozygosity of the cattle markers in buffalo was 0.52, but in general the average heterozygosity of these markers is significantly higher on cattle populations [9,14]. The average heterozygosity of the newly isolated buffalo markers was significantly higher (0.62) than those of cattle markers in buffalo. It could be due to selection of microsatellite markers with high number of repeats from buffalo genomic library which in turn is likely to be associated with high level of polymorphism for these markers in buffalo. There is no option for such kind of selection when the markers are used in related species [15]. Out of 498 polymorphic loci, 56 loci showed significant (P < 0.05) departure from Hardy-Weinberg equilibrium after applying bonferroni correction, reflecting an excess of homozygous individuals in the population. Several hypotheses have been mentioned to explain homozygote excess, including inbreeding, population admixture and null alleles. However, null allele is a usually referred one for homozygote excess in many cases. Therefore, null allele presence was checked at each locus, notably 79% of the loci that deviated from HWE showed null allele presence in the cattle markers whereas this value was 62% for buffalo markers.

**Figure 4:**
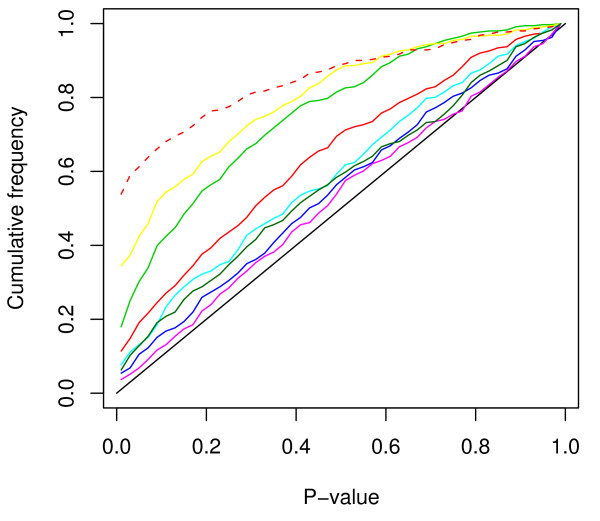
**Cumulative frequency distribution plot**. The cumulative frequency distribution of LD P-value obtained between 27 polymorphic microsatellite markers on eight different buffalo breeds; Bhadawari (red line), Jaffarabadi (light green), Mehsana (blue line), Murrah (light blue line), Nagpuri (magenta line), Pandharpuri (yellow line), Surati (green line), Toda (red dashed line). The black line represents the expected distribution under random allelic segregation.

Characterization of LD between the markers provides insights to assess the power of association studies to map the loci underlying traits of interest. In the present study, large number of pairs exhibited significant LD for buffalo markers than that for cattle markers. It may be due to high heterozygosity and the presence of null alleles at high frequency in the buffalo markers. Out of 418 cattle derived markers, 55 (14%) showed null allele presence whereas 23% of buffalo markers showed null allele presence. At the same time in the absence of mapping data for these loci, we cannot rule out the possibility that the closely linked markers (syntenic markers) would exhibit higher LD. It has been showed in many LD studies that the P-values obtained from the test of significant departure from LD between the loci mainly depended on the sample size [16]. Therefore, to find out the effect of the sample size on LD, we used different datasets. First we analyzed LD on a dataset containing genotypic information of 24 Murrah buffalos for 27 highly polymorphic makers, 8.8% of these marker pairs showed significant LD (P < 0.05). Subsequently, we increased the sample size from 24 to 48 for the same 27 markers and checked the LD; now 11.4% marker pairs showed significant (P < 0.05) LD. Thus, sample size did not affect the LD level to a large extent in the present study. Furthermore, we checked the LD for these 27 markers on eight well-recognized Indian water buffalo breeds [17]. Figure 4 shows the cumulative frequency distribution of Fisher's exact test P-values of eight buffalo breeds. There was a large difference between the breeds in the number of pairs showing significant LD (Table 1). Comparatively large numbers of pairs with high LD were observed in Toda, Pandharpuri and Jaffarabadi breeds. It has been shown that rapidly growing populations show less LD as compared to constant size populations [18]. Although breed wise census data are not available, the number of Toda animals has been declining sharply [17], and thus, it is not surprising to find high number of markers pairs showing significant LD in this breed.

**Table 1:** Fisher's exact test P-values for linkage disequilibrium for 27 microsatellite markers genotyped on eight different buffalo breeds.

**Markers used**	**Buffalo breeds**	**Sample size**	**No. of marker pairs significant at P < 0.05**
BMS4012, BMS518	Bhadawari	48	60 (17.1%)
	
CA004, TGLA36	Jaffarabadi	47	95 (27.1%)
	
BMS1316, BMS1724 BMS4016, BMS462	Mehsana	48	32 (9.1%)
	
BM757, BMS2519 ILSTS058, TGLA159	Murrah	48	40 (11.4%)
	
BL1029, BM1352 BMS2325, BMS2847	Nagpuri	48	20 (5.7%)
	
BM4513, CSSM047 ILSTS089, BL1036	Pandharpuri	48	142 (40.4%)
	
BMS2116, MSBQ RM372, AFR227	Surati	48	39 (11.1%)
	
BL1134, BMS1226 BMS1747	Toda	48	208 (59.3%)

## Conclusion

Till date no comprehensive genome mapping efforts have been devoted to water buffalo. Hence, to develop a microsatellite based linkage map of water buffalo, we have been evaluating genetic markers for water buffalo and here we have reported the characteristics of 571 microsatellite markers. Further to check their applicability in radiation hybrid map of water buffalo, these markers were tested on Chinese hamster genomic DNA for amplification, only seven markers showed amplification in Chinese hamster [see Additional file 3] suggesting that the rest of the 564 markers would be immediately useful for defining their position on a radiation hybrid map of buffalo genome. These 498 polymorphic markers will be very useful in population genetic studies and for genetic dissection of complex traits in buffalo. At the same time, the newly developed buffalo markers can be tested on other bovidae species to amplify corresponding loci.

## Methods

### Microsatellite markers development

To develop microsatellite markers for buffalo genome, we used comparative genomics approach. 594 cattle microsatellite markers distributed across 23 chromosomes were chosen to test on water buffalo. All the markers and primers details were obtained from , BOVMAP and Bishop et al [5]. Additional buffalo microsatellite markers were isolated through a small genomic library construction using the standard protocol described previously [19]. Genomic DNA was extracted from a Murrah buffalo blood sample by phenol-chloroform method [20]. PRIMER 3.0 software [21] was used to design the specific primer sets.

### Validation of microsatellite markers

By these two approaches, we got a total of 803 microsatellite markers and tested them for amplification on buffalo genome. The PCR reaction was carried out in a total volume of 10 μl using 50 ng template DNA, 1 pM of each primer and AmpliTaq Gold PCR master mix (Applied Biosystems, Roche Molecular Systems, Inc.). Polymerase chain reactions were performed using Mastercycler (Eppendorf) and GeneAmp^® ^PCR System 9700 (Applied Biosystems) under the following conditions: an initial denaturation at 95°C for 5 min followed by 30 cycles at 94°C for 1 min, respective primer annealing temperature for 45 sec and 72°C for 1 min. A final elongation step of 7 min was carried out at 72°C. The PCR products were visualized on 2% agarose gel. Primers presenting discrete bands with expected size were further tested on a panel of 24 unrelated Murrah buffaloes. Murrah animals with typical phenotypic features have collected from different places from Haryana state (India). The amplified PCR products were multiplexed, for multiplex development the forward primers were labelled with four different fluorescent dyes (FAM, VIC, PET, NED) supplied by Applied Biosystems (Roche Molecular Systems, Inc. USA). Genotyping was done using ABI 3730 automated DNA sequencer. To avoid having false negative results due to PCR artefacts, instability at a locus was scored only when microsatellite alterations could be reproducible in repeat PCR reactions.

### Data analysis

GENEMAPPER version 3.5 (Applied Biosystem) was used to resolve the microsatellite allele size. The number of alleles, observed heterozygosity (Ho) and expected heterozygosity (He) per locus were estimated using the software MICROSATELLITE ANALYSER (MSA) version 3.15 [22]. Hardy-Weinberg equilibrium of the each loci were tested using an exact test implemented in the GENEPOP software [23]. The exact linkage disequilibrium P values for the observed allelic association under the null hypothesis of random allelic assortment were estimated by Markov chain-Monte Carlo algorithm using ARLEQUIN software [24]. Also, In order to determine the effect of sample size on LD, we used different datasets, and only 27 highly polymorphic cattle derived markers were selected to use (Table 1). Because it has been observed by in this study that, heterozygosity positively correlated with LD on the other side the heterozygosity level of the cattle markers slightly lower than the buffalo markers, so that the outcome of the study can easily compare with previous reports. The probability of null alleles at each locus was calculated using MICRO CHECKER [25]. The plots were drawn using R software .

## Authors' contributions

SK conceived and designed the study; NK, GN, RH, KP, JG, MM, RV, ShK, and AR performed the experiments; MN analyzed the data; MN and SK wrote the paper. All authors read and approved the final manuscript.

## Supplementary Material

Additional file 1 Characteristics of polymorphic microsatellite loci of Bubalus bubalis developed through cross species amplification. All these markers were originally developed for cattle. Click here for file

Additional file 2 Monomorphic microsatellite loci of Bubalus bubalis developed through cross species amplification. All these markers were originally developed for cattle. Click here for file

Additional file 3 Characteristics of polymorphic microsatellite loci developed from an enriched genomic library of Bubalus bubalis. Click here for file

Additional file 4 Monomorphic microsatellite loci derived from an enriched genomic library of Bubalus bubalis. Click here for file
