# Supplementary material for: Microsatellite markers of water buffalo, Bubalus bubalis - development, characterisation and linkage disequilibrium studies
Source: BMC Genet. 2009 Oct 21;10:68. doi: 10.1186/1471-2156-10-68 (PMC2773805; doi:10.1186/1471-2156-10-68)
Supplement: Additional file 1 — Characteristics of polymorphic microsatellite loci of Bubalus bubalis developed through cross species amplification. All these markers were originally developed for cattle. [file 1471-2156-10-68-S1.DOC]

**Characteristics of polymorphic microsatellite loci of *Bubalus bubalis* developed through cross species amplification. All these markers were originally developed for cattle.**

| S. No | Locus | No. of. Alleles | Allele size | He. obs | He. exp |  |
| --- | --- | --- | --- | --- | --- | --- |
|  |  |  |  |  |  | Null allele presence |
| 1 | ACC08 | 7 | 120-138 | 0.625 | 0.69 | No |
| 2 | ACO1 | 6 | 115-149 | 0.333 | 0.818 | Yes |
| 3 | AFR227 | 5 | 101-121 | 0.9 | 0.764 | No |
| 4 | AGLA232 | 4 | 157-163 | 0.695 | 0.575 | No |
| 5 | BB1542 | 3 | 198-212 | 0.375 | 0.411 | No |
| 6 | BB709 | 3 | 203-213 | 0.272 | 0.246 | No |
| 7 | BB718 | 5 | 151-177 | 0.772 | 0.778 | No |
| 8 | BL042 | 4 | 239-255 | 0.636 | 0.687 | No |
| 9 | BL1006 | 2 | 113-115 | 0.666 | 0.483 | No |
| 10 | BL1009 | 2 | 163-165 | 0.083 | 0.283 | Yes |
| 11 | BL1024 | 7 | 103-121 | 0.727 | 0.688 | No |
| 12 | BL1028 | 6 | 10-103 | 0.857 | 0.746 | No |
| 13 | BL1029 | 5 | 144-158 | 0.818 | 0.769 | No |
| 14 | BL1030 | 2 | 147-155 | 0.571 | 0.483 | No |
| 15 | BL1036 | 6 | 177-191 | 0.857 | 0.811 | No |
| 16 | BL1038 | 6 | 103-113 | 0.631 | 0.714 | No |
| 17 | BL1067 | 5 | 88-96 | 1 | 0.723 | No |
| 18 | BL1071 | 3 | 179-185 | 0.523 | 0.451 | No |
| 19 | BL1095 | 4 | 147-173 | 0.782 | 0.744 | No |
| 20 | BL1103 | 3 | 107-113 | 0.681 | 0.647 | No |
| 21 | BL1134 | 5 | 100-118 | 0.458 | 0.688 | Yes |
| 22 | BL25 | 5 | 192-202 | 0.368 | 0.48 | No |
| 23 | BL28 | 2 | 113-115 | 0.333 | 0.507 | No |
| 24 | BL37 | 3 | 242-260 | 0.6 | 0.509 | No |
| 25 | BL4 | 5 | 132-154 | 0.59 | 0.51 | No |
| 26 | BL41 | 3 | 245-249 | 0.304 | 0.426 | No |
| 27 | BM045 | 3 | 138-156 | 0.833 | 0.538 | No |
| 28 | BM121 | 9 | 131-163 | 0.733 | 0.783 | No |
| 29 | BM1258 | 4 | 102-120 | 0.130* | 0.688 | Yes |
| 30 | BM1311 | 4 | 124-138 | 0.619 | 0.702 | No |
| 31 | BM1443 | 6 | 123-139 | 0.714 | 0.785 | No |
| 32 | BM148 | 4 | 102-112 | 0.2 | 0.488 | Yes |
| 33 | BM1508 | 5 | 98-124 | 0.666 | 0.573 | No |
| 34 | BM17052 | 4 | 204-212 | 0.8 | 0.583 | No |
| 35 | BM1706 | 9 | 235-261 | 0.809 | 0.871 | No |
| 36 | BM17132 | 7 | 77-93 | 0.826 | 0.736 | No |
| 37 | BM1815 | 5 | 226-250 | 0.437 | 0.54 | No |
| 38 | BM1827 | 3 | 157-169 | 0 | 0.392 | Yes |
| 39 | BM1853 | 4 | 103-113 | 0.809 | 0.761 | No |
| 40 | BM1861 | 11 | 103-125 | 1 | 0.875 | No |
| 41 | BM1862 | 5 | 202-214 | 0.250* | 0.592 | Yes |
| 42 | BM203 | 4 | 222-234 | 0.545 | 0.607 | No |
| 43 | BM2607 | 4 | 138-144 | 0.5 | 0.507 | No |
| 44 | BM2808 | 2 | 146-148 | 0 | 0.102 | No |
| 45 | BM2934 | 3 | 89-103 | 0.75 | 0.549 | No |
| 46 | BM310 | 4 | 130-144 | 0.526 | 0.439 | No |
| 47 | BM315 | 4 | 102-108 | 0.444 | 0.498 | No |
| 48 | BM3419 | 2 | 133-135 | 0.095 | 0.092 | No |
| 49 | BM3501 | 6 | 167-187 | 0.705 | 0.691 | No |
| 50 | BM3507 | 5 | 160-178 | 0.5 | 0.575 | No |
| 51 | BM3509 | 5 | 102-114 | 0.59 | 0.7 | No |
| 52 | BM4006 | 2 | 102-114 | 0.21 | 0.193 | No |
| 53 | BM4028 | 7 | 116-144 | 0.318 | 0.745 | Yes |
| 54 | BM4208 | 7 | 162-178 | 0.875 | 0.829 | No |
| 55 | BM4301 | 5 | 128-136 | 0.736 | 0.8 | No |
| 56 | BM4305 | 4 | 146-154 | 0.708 | 0.705 | No |
| 57 | BM4311 | 6 | 85-107 | 0.45 | 0.428 | No |
| 58 | BM4509 | 5 | 126-146 | 0.5 | 0.612 | No |
| 59 | BM4513 | 5 | 128-144 | 0.863 | 0.804 | No |
| 60 | BM5004 | 2 | 109-119 | 0.45 | 0.357 | No |
| 61 | BM6105 | 6 | 84-97 | 0.833 | 0.747 | No |
| 62 | BM6108 | 3 | 117-13 | 0.352 | 0.313 | No |
| 63 | BM6117 | 7 | 114-136 | 0.782 | 0.825 | No |
| 64 | BM6121 | 4 | 148-156 | 0.000* | 0.641 | Yes |
| 65 | BM6122 | 6 | 82-102 | 0.739 | 0.716 | No |
| 66 | BM6404 | 5 | 133-149 | 0.761 | 0.666 | No |
| 67 | BM6418 | 3 | 156-168 | 0.583 | 0.467 | No |
| 68 | BM6425 | 2 | 193-196 | 0.541 | 0.403 | No |
| 69 | BM6437 | 6 | 232-242 | 0.809 | 0.789 | No |
| 70 | BM6438 | 3 | 259-269 | 0.388 | 0.341 | No |
| 71 | BM6444 | 3 | 83-87 | 0.125 | 0.263 | Yes |
| 72 | BM6445 | 7 | 140-156 | 0.421 | 0.694 | Yes |
| 73 | BM6449 | 5 | 136-156 | 0.625 | 0.645 | No |
| 74 | BM6458 | 2 | 105-115 | 0.142 | 0.135 | No |
| 75 | BM6548 | 8 | 241-259 | 0.666 | 0.729 | No |
| 76 | BM7145 | 2 | 119-121 | 0.086 | 0.085 | No |
| 77 | BM7169 | 4 | 221-229 | 0.318 | 0.449 | No |
| 78 | BM719 | 4 | 126-153 | 0.05 | 0.191 | Yes |
| 79 | BM720 | 4 | 223-231 | 0.217 | 0.408 | No |
| 80 | BM7208 | 3 | 136-168 | 0.937 | 0.542 | yes |
| 81 | BM7234 | 3 | 118-122 | 0.5 | 0.509 | No |
| 82 | BM7247 | 6 | 98-138 | 0.235* | 0.759 | Yes |
| 83 | BM746 | 3 | 149-153 | 0.652 | 0.584 | No |
| 84 | BM757 | 9 | 189-208 | 0.875 | 0.782 | No |
| 85 | BM8115 | 2 | 131-135 | 0.318 | 0.332 | No |
| 86 | BM8124 | 3 | 105-109 | 0.65 | 0.626 | No |
| 87 | BM8126 | 10 | 133-152 | 0.791 | 0.804 | No |
| 88 | BM860 | 2 | 175-177 | 0.272 | 0.494 | No |
| 89 | BM871 | 3 | 138-142 | 0.25 | 0.383 | No |
| 90 | BM888 | 8 | 173-201 | 0.85 | 0.823 | No |
| 91 | BM9034 | 6 | 129-153 | 0.421 | 0.743 | Yes |
| 92 | BM9138 | 3 | 166-170 | 0.227 | 0.474 | Yes |
| 93 | BM9248 | 2 | 230-234 | 0.347 | 0.293 | No |
| 94 | BMC1009 | 4 | 280-300 | 0.666 | 0.687 | No |
| 95 | BMC1013 | 7 | 223-247 | 0.869 | 0.807 | No |
| 96 | BMC1207 | 2 | 125-127 | 0.26 | 0.486 | No |
| 97 | BMC4203 | 9 | 156-171 | 0.904 | 0.839 | No |
| 98 | BMC6020 | 3 | 168-180 | 0.857 | 0.669 | No |
| 99 | BMS1001 | 3 | 138-144 | 0.7 | 0.678 | No |
| 100 | BMS1004 | 4 | 142-148 | 0.708 | 0.613 | No |
| 101 | BMS1069 | 5 | 104-116 | 0.681 | 0.601 | No |
| 102 | BMS1074 | 6 | 157-169 | 0.583 | 0.57 | No |
| 103 | BMS108 | 2 | 88-90 | 0.59 | 0.459 | No |
| 104 | BMS1095 | 6 | 99-119 | 0.666 | 0.585 | No |
| 105 | BMS1101 | 6 | 170-188 | 0.652 | 0.622 | No |
| 106 | BMS1148 | 3 | 168-174 | 0.166 | 0.227 | No |
| 107 | BMS1185 | 2 | 135-137 | 0.238 | 0.214 | No |
| 108 | BMS119 | 6 | 121-139 | 0.272* | 0.674 | Yes |
| 109 | BMS1216 | 5 | 142-154 | 0.684 | 0.722 | No |
| 110 | BMS1226 | 7 | 154-174 | 0.863 | 0.778 | No |
| 111 | BMS1231 | 7 | 97-113 | 0.809 | 0.688 | No |
| 112 | BMS1234 | 5 | 132-146 | 0.434 | 0.551 | No |
| 113 | BMS1248 | 8 | 118-141 | 0.65 | 0.762 | No |
| 114 | BMS1266 | 5 | 121-133 | 0.538 | 0.784 | No |
| 115 | BMS1290 | 4 | 92-106 | 0.45 | 0.644 | No |
| 116 | BMS1304 | 7 | 120-138 | 0.521 | 0.6 | No |
| 117 | BMS1316 | 6 | 113-129 | 0.714 | 0.766 | No |
| 118 | BMS1318 | 2 | 143-147 | 0.608 | 0.51 | No |
| 119 | BMS1322 | 3 | 134-140 | 0.277 | 0.398 | No |
| 120 | BMS1331 | 7 | 141-157 | 0.687 | 0.814 | No |
| 121 | BMS1348 | 2 | 112-127 | 0.238 | 0.214 | No |
| 122 | BMS1352 | 7 | 99-115 | 0.636 | 0.8 | No |
| 123 | BMS14 | 8 | 102-122 | 0.791 | 0.878 | No |
| 124 | BMS1494 | 3 | 105-109 | 0.173 | 0.239 | No |
| 125 | BMS1600 | 7 | 142-184 | 0.75 | 0.698 | No |
| 126 | BMS1617 | 3 | 167-171 | 0.142 | 0.138 | No |
| 127 | BMS1620 | 6 | 86-102 | 0.391 | 0.383 | No |
| 128 | BMS1675 | 5 | 82-92 | 0.4 | 0.591 | No |
| 129 | BMS1676 | 8 | 140-174 | 0.772 | 0.784 | No |
| 130 | BMS1678 | 4 | 130-144 | 0.541 | 0.651 | No |
| 131 | BMS1714 | 3 | 112-127 | 0.238 | 0.219 | No |
| 132 | BMS1716 | 7 | 204-220 | 0.625 | 0.677 | No |
| 133 | BMS1724 | 6 | 149-173 | 0.434 | 0.776 | Yes |
| 134 | BMS1742 | 6 | 165-175 | 0.818 | 0.705 | No |
| 135 | BMS1747 | 9 | 91-107 | 0.772 | 0.761 | No |
| 136 | BMS1758 | 4 | 153-163 | 0.619 | 0.663 | No |
| 137 | BMS1787 | 5 | 151-165 | 0.636 | 0.608 | No |
| 138 | BMS1789 | 5 | 107-121 | 0.5 | 0.642 | No |
| 139 | BMS1822 | 2 | 85-87 | 0.588 | 0.427 | No |
| 140 | BMS1825 | 3 | 136-160 | 0.722 | 0.565 | No |
| 141 | BMS1840 | 4 | 180-190 | 0.722 | 0.755 | No |
| 142 | BMS1902 | 7 | 134-162 | 0.375* | 0.81 | Yes |
| 143 | BMS1909 | 2 | 79-85 | 0.687 | 0.465 | No |
| 144 | BMS1915 | 3 | 79-83 | 0.388 | 0.331 | No |
| 145 | BMS1920 | 5 | 110-121 | 0.578 | 0.58 | No |
| 146 | BMS1928 | 4 | 149-157 | 0.6 | 0.557 | No |
| 147 | BMS1939 | 4 | 115-129 | 0.5 | 0.569 | No |
| 148 | BMS1943 | 5 | 123-145 | 0.6 | 0.616 | No |
| 149 | BMS1979 | 7 | 88-112 | 0.625 | 0.731 | No |
| 150 | BMS1987 | 2 | 123-127 | 0.166 | 0.156 | No |
| 151 | BMS2047 | 5 | 135-149 | 0.523 | 0.701 | No |
| 152 | BMS2055 | 2 | 145-147 | 0.391 | 0.414 | No |
| 153 | BMS2060 | 5 | 89-99 | 0.625 | 0.564 | No |
| 154 | BMS2063 | 4 | 104-114 | 0.208 | 0.267 | No |
| 155 | BMS2072 | 3 | 164-172 | 0.526 | 0.465 | No |
| 156 | BMS2079 | 6 | 89-107 | 0.583 | 0.476 | No |
| 157 | BMS2104 | 2 | 159-161 | 0.545 | 0.443 | No |
| 158 | BMS2116 | 6 | 102-120 | 0.565 | 0.705 | No |
| 159 | BMS2137 | 3 | 123-127 | 0.565 | 0.456 | No |
| 160 | BMS2142 | 6 | 88-104 | 0.736 | 0.776 | No |
| 161 | BMS2149 | 4 | 105-111 | 0.260* | 0.555 | Yes |
| 162 | BMS2208 | 6 | 101-111 | 0.692 | 0.753 | No |
| 163 | BMS2252 | 5 | 165-177 | 0.708 | 0.612 | No |
| 164 | BMS2258 | 3 | 123-143 | 0.428 | 0.47 | No |
| 165 | BMS2263 | 3 | 164-168 | 0.523 | 0.535 | No |
| 166 | BMS2275 | 2 | 113-115 | 0.25 | 0.387 | No |
| 167 | BMS2295 | 4 | 112-122 | 0.478 | 0.673 | Yes |
| 168 | BMS2319 | 3 | 105-113 | 0.652 | 0.613 | No |
| 169 | BMS2325 | 5 | 120-128 | 0.833 | 0.781 | No |
| 170 | BMS2377 | 7 | 104-144 | 1 | 0.738 | No |
| 171 | BMS2466 | 2 | 62-66 | 0.3 | 0.261 | No |
| 172 | BMS2503 | 7 | 160-178 | 0.647 | 0.832 | No |
| 173 | BMS2513 | 6 | 167-183 | 0.652 | 0.697 | No |
| 174 | BMS2519 | 5 | 104-118 | 0.75 | 0.737 | No |
| 175 | BMS2572 | 5 | 96-106 | 0.375 | 0.523 | No |
| 176 | BMS2608 | 5 | 143-151 | 0.913 | 0.718 | No |
| 177 | BMS2629 | 3 | 147-155 | 0.277 | 0.538 | Yes |
| 178 | BMS2641 | 4 | 163-179 | 0.739 | 0.59 | No |
| 179 | BMS2646 | 2 | 123-129 | 0.142 | 0.135 | No |
| 180 | BMS2724 | 3 | 73-77 | 0.391 | 0.583 | No |
| 181 | BMS2742 | 6 | 134-152 | 0.8 | 0.761 | No |
| 182 | BMS2753 | 3 | 106-110 | 0.631 | 0.621 | No |
| 183 | BMS2780 | 4 | 128-150 | 0.708 | 0.543 | No |
| 184 | BMS2809 | 2 | 137-139 | 0.19 | 0.25 | No |
| 185 | BMS2847 | 5 | 206-216 | 0.714 | 0.777 | No |
| 186 | BMS3002 | 5 | 148-156 | 0.578 | 0.662 | No |
| 187 | BMS356 | 5 | 86-104 | 0.428 | 0.626 | Yes |
| 188 | BMS357 | 6 | 101-121 | 0.818 | 0.728 | No |
| 189 | BMS4000 | 6 | 124-140 | 0.85 | 0.673 | No |
| 190 | BMS4006 | 4 | 101-113 | 0.142* | 0.537 | Yes |
| 191 | BMS4011 | 4 | 199-205 | 0.608 | 0.567 | No |
| 192 | BMS4012 | 6 | 96-105 | 0.291* | 0.724 | Yes |
| 193 | BMS4013 | 8 | 78-100 | 0.944 | 0.857 | No |
| 194 | BMS4016 | 7 | 129-157 | 0.956 | 0.757 | No |
| 195 | BMS4021 | 2 | 124-126 | 0.521 | 0.486 | No |
| 196 | BMS4024 | 5 | 178-198 | 0.59 | 0.7 | No |
| 197 | BMS4030 | 3 | 153-165 | 0.388 | 0.414 | No |
| 198 | BMS4036 | 2 | 91-99 | 0.222 | 0.203 | No |
| 199 | BMS4049 | 4 | 97-109 | 0.478 | 0.39 | No |
| 200 | BMS4050 | 4 | 152-164 | 0.176* | 0.611 | Yes |
| 201 | BMS4052 | 3 | 170-179 | 0.478 | 0.543 | No |
| 202 | BMS410 | 3 | 75-91 | 0.333 | 0.493 | No |
| 203 | BMS417 | 6 | 111-133 | 0.473 | 0.733 | Yes |
| 204 | BMS425 | 8 | 96-122 | 0.863 | 0.788 | No |
| 205 | BMS429 | 4 | 134-140 | 0.588 | 0.641 | No |
| 206 | BMS434 | 5 | 103-125 | 0.583 | 0.652 | No |
| 207 | BMS460 | 4 | 115-121 | 0.521 | 0.422 | No |
| 208 | BMS462 | 7 | 118-136 | 0.772 | 0.792 | No |
| 209 | BMS47 | 7 | 139-150 | 0.772 | 0.835 | No |
| 210 | BMS483 | 2 | 120-122 | 0.086 | 0.085 | No |
| 211 | BMS499 | 5 | 110-132 | 0.666 | 0.664 | No |
| 212 | BMS510 | 3 | 97-101 | 0.727 | 0.531 | No |
| 213 | BMS511 | 6 | 107-119 | 0.739 | 0.761 | No |
| 214 | BMS518 | 6 | 152-166 | 0.782 | 0.714 | No |
| 215 | BMS528 | 4 | 151-161 | 0.583 | 0.632 | No |
| 216 | BMS538 | 2 | 112-127 | 0.111 | 0.111 | No |
| 217 | BMS555 | 4 | 178-184 | 0.409 | 0.612 | No |
| 218 | BMS585 | 5 | 121-135 | 0.59 | 0.567 | No |
| 219 | BMS597 | 9 | 151-170 | 0.826 | 0.834 | No |
| 220 | BMS599 | 6 | 113-123 | 0.772 | 0.737 | No |
| 221 | BMS607 | 2 | 136-138 | 0.095 | 0.455 | Yes |
| 222 | BMS614 | 4 | 136-144 | 0.772 | 0.643 | No |
| 223 | BMS648 | 5 | 155-161 | 0.7 | 0.634 | No |
| 224 | BMS649 | 7 | 140-156 | 0.5 | 0.718 | Yes |
| 225 | BMS678 | 8 | 104-124 | 1 | 0.822 | No |
| 226 | BMS711 | 6 | 105-119 | 0.478 | 0.552 | No |
| 227 | BMS719 | 2 | 92-98 | 0.333 | 0.48 | No |
| 228 | BMS739 | 8 | 101-127 | 0.833 | 0.815 | No |
| 229 | BMS745 | 6 | 107-121 | 0.5 | 0.644 | No |
| 230 | BMS772 | 6 | 131-151 | 0.611 | 0.723 | No |
| 231 | BMS812 | 4 | 82-94 | 0.625 | 0.515 | No |
| 232 | BMS813 | 5 | 205-215 | 0.625 | 0.687 | No |
| 233 | BMS817 | 4 | 120-126 | 0.291 | 0.424 | Yes |
| 234 | BMS827 | 5 | 101-115 | 0.533 | 0.678 | No |
| 235 | BMS829 | 6 | 120-134 | 0.727 | 0.724 | No |
| 236 | BMS861 | 2 | 147-149 | 0.347 | 0.347 | No |
| 237 | BMS862 | 5 | 136-156 | 0.437 | 0.63 | No |
| 238 | BMS885 | 4 | 162-168 | 0.454 | 0.63 | No |
| 239 | BMS887 | 3 | 148-162 | 0.529 | 0.504 | No |
| 240 | BMS922 | 8 | 74-92 | 0.809 | 0.838 | No |
| 241 | BMS927 | 2 | 174-176 | 0 | 0.102 | No |
| 242 | BMS941 | 8 | 80-104 | 0.8 | 0.817 | No |
| 243 | BMS963 | 4 | 153-159 | 0.904 | 0.695 | No |
| 244 | BMS975 | 3 | 80-90 | 0.25 | 0.429 | Yes |
| 245 | BMS995 | 6 | 135-145 | 0.739 | 0.705 | No |
| 246 | BP031 | 2 | 196-198 | 0.083 | 0.081 | No |
| 247 | BP1 | 2 | 277-283 | 0.217 | 0.198 | No |
| 248 | BP2 | 2 | 359-361 | 0.086 | 0.085 | No |
| 249 | BP20 | 7 | 224-258 | 0.300* | 0.75 | Yes |
| 250 | BP23 | 2 | 258-260 | 0.176 | 0.336 | No |
| 251 | BP34 | 6 | 300-318 | 0.636 | 0.633 | No |
| 252 | BP7 | 2 | 296-297 | 0.000* | 0.496 | Yes |
| 253 | BR1603 | 4 | 143-151 | 0.4 | 0.752 | Yes |
| 254 | BR2724 | 2 | 154-156 | 0.09 | 0.246 | No |
| 255 | BR2936 | 7 | 144-164 | 0.809 | 0.677 | No |
| 256 | BR3510 | 7 | 96-112 | 0.727 | 0.743 | No |
| 257 | BR6504 | 4 | 112-130 | 0.809 | 0.679 | No |
| 258 | BY1504 | 6 | 92-116 | 0.565 | 0.545 | No |
| 259 | CA004 | 9 | 138-168 | 0.75 | 0.748 | No |
| 260 | CA028 | 6 | 114-132 | 0.555 | 0.709 | No |
| 261 | CA067 | 2 | 92-94 | 0.529 | 0.45 | No |
| 262 | CA088 | 6 | 115-149 | 0.333* | 0.818 | Yes |
| 263 | CA090 | 4 | 124-132 | 0.411 | 0.356 | No |
| 264 | CA095 | 5 | 118-132 | 0.708 | 0.74 | No |
| 265 | CA096 | 4 | 102-112 | 0.227 | 0.288 | No |
| 266 | CA099 | 4 | 93-107 | 0.208* | 0.507 | Yes |
| 267 | CA209 | 3 | 106-109 | 0.55 | 0.644 | No |
| 268 | CP026 | 2 | 116-122 | 0.35 | 0.296 | No |
| 269 | CSSM014 | 3 | 135-139 | 0.095 | 0.256 | Yes |
| 270 | CSSM019 | 5 | 136-156 | 0.526 | 0.692 | No |
| 271 | CSSM024 | 2 | 105-107 | 0.363 | 0.443 | No |
| 272 | CSSM033 | 6 | 160-178 | 0.625 | 0.587 | No |
| 273 | CSSM034 | 7 | 98-114 | 0.904 | 0.804 | No |
| 274 | CSSM036 | 5 | 170-184 | 0.652 | 0.681 | No |
| 275 | CSSM037 | 4 | 183-191 | 0.541 | 0.681 | No |
| 276 | CSSM038 | 3 | 167-189 | 0.454 | 0.512 | No |
| 277 | CSSM039 | 5 | 179-187 | 0.458 | 0.505 | No |
| 278 | CSSM043 | 4 | 226-258 | 0.571 | 0.706 | No |
| 279 | CSSM046 | 3 | 158-166 | 0.043 | 0.273 | Yes |
| 280 | CSSM047 | 9 | 138-172 | 0.647 | 0.823 | No |
| 281 | EL03 | 3 | 150-154 | 0.521 | 0.513 | No |
| 282 | ETH07 | 4 | 370-384 | 0.136 * | 0.455 | Yes |
| 283 | ETH2 | 5 | 162-172 | 0.416 | 0.455 | No |
| 284 | FCB11 | 9 | 130-142 | 0.708* | 0.811 | No |
| 285 | FCB48 | 5 | 148-174 | 0.666 | 0.688 | No |
| 286 | HAUT1 | 2 | 149-151 | 0.347 | 0.347 | No |
| 287 | HU414 | 7 | 161-177 | 0.818 | 0.753 | No |
| 288 | HUJ1177 | 5 | 200-226 | 0.5 | 0.518 | No |
| 289 | HUJ223 | 3 | 169-179 | 0.473 | 0.594 | No |
| 290 | HUJ614 | 3 | 174-180 | 0.363 | 0.368 | No |
| 291 | HUJ616 | 4 | 115-135 | 0.375 | 0.419 | No |
| 292 | HUJ625 | 2 | 198-206 | 0.235 | 0.213 | No |
| 293 | HUJV174 | 8 | 139-177 | 0.857 | 0.849 | No |
| 294 | IBSP | 2 | 159-177 | 1.000* | 0.512 | No |
| 295 | IDVGA027 | 3 | 151-155 | 0.333 | 0.426 | No |
| 296 | IDVGA03 | 5 | 166-178 | 0.565 | 0.671 | No |
| 297 | IDVGA11 | 3 | 253-273 | 0.875 | 0.62 | No |
| 298 | IDVGA2 | 4 | 138-146 | 0.454 | 0.569 | No |
| 299 | IDVGA29 | 3 | 127-135 | 0.388 | 0.331 | No |
| 300 | IDVGA32 | 8 | 208-226 | 0.863 | 0.808 | No |
| 301 | IDVGA37 | 5 | 182-194 | 0.578 | 0.736 | No |
| 302 | IDVGA40 | 6 | 241-255 | 0.666 | 0.687 | No |
| 303 | IDVGA64 | 5 | 219-249 | 0.578 | 0.541 | No |
| 304 | ILSTS080 | 3 | 97-103 | 0.318 | 0.457 | No |
| 305 | ILSTS083 | 3 | 204-210 | 0.619 | 0.585 | No |
| 306 | ILSTS100 | 2 | 198-200 | 0.227 | 0.459 | Yes |
| 307 | INRA050 | 2 | 136-178 | 0.954* | 0.51 | No |
| 308 | INRA084 | 6 | 92-116 | 0.684 | 0.65 | No |
| 309 | INRA092 | 4 | 138-152 | 0.478 | 0.59 | No |
| 310 | INRA096 | 5 | 117-137 | 0.521 | 0.541 | No |
| 311 | INRA100 | 4 | 157-171 | 0.5 | 0.59 | No |
| 312 | INRA112 | 7 | 170-188 | 0.956 | 0.825 | No |
| 313 | INRA119 | 5 | 122-130 | 0.739 | 0.702 | No |
| 314 | INRA132 | 3 | 150-160 | 0 | 0.189 | Yes |
| 315 | INRA133 | 7 | 226-242 | 1 | 0.806 | No |
| 316 | INRA134 | 7 | 131-145 | 0.833 | 0.718 | No |
| 317 | INRA135 | 4 | 112-126 | 0.708 | 0.619 | No |
| 318 | INRA144 | 5 | 161-175 | 0.708 | 0.752 | No |
| 319 | INRA162 | 4 | 93-113 | 0.454 | 0.538 | No |
| 320 | INRA177 | 3 | 78-88 | 0.38 | 0.329 | No |
| 321 | INRA183 | 7 | 129-153 | 0.705 | 0.631 | No |
| 322 | INRA192 | 6 | 140-160 | 0.8 | 0.749 | No |
| 323 | INRA193 | 8 | 145-167 | 0.416 | 0.756 | Yes |
| 324 | JAB1 | 6 | 234-244 | 0.708 | 0.726 | No |
| 325 | JAB8 | 2 | 177-179 | 0 | 0.081 | Yes |
| 326 | MAF050 | 7 | 153-167 | 1 | 0.827 | No |
| 327 | MAF23 | 5 | 119-133 | 0.666 | 0.723 | No |
| 328 | MB008 | 8 | 237-253 | 0.521 | 0.788 | Yes |
| 329 | MB010 | 8 | 236-256 | 0.882 | 0.759 | No |
| 330 | MB019 | 5 | 186-194 | 0.583 | 0.679 | No |
| 331 | MB022 | 5 | 198-222 | 0.333 | 0.59 | Yes |
| 332 | MB026 | 8 | 224-242 | 0.8 | 0.795 | No |
| 333 | MB055 | 3 | 96-104 | 0.111 | 0.109 | No |
| 334 | MB058 | 5 | 212-224 | 0.454* | 0.674 | Yes |
| 335 | MB062 | 2 | 212-214 | 0.136 | 0.51 | Yes |
| 336 | MB064 | 2 | 154-156 | 1.000* | 0.511 | No |
| 337 | MB076 | 2 | 176-178 | 0.958* | 0.509 | No |
| 338 | MB077 | 6 | 240-252 | 0.625 | 0.523 | No |
| 339 | MB085 | 5 | 204-220 | 0.727 | 0.705 | No |
| 340 | MB099 | 2 | 191-193 | 0.083 | 0.081 | No |
| 341 | MB106 | 7 | 226-272 | 0.842 | 0.793 | No |
| 342 | MCM64 | 2 | 136-138 | 0.055 | 0.055 | No |
| 343 | MSBQ | 6 | 122-138 | 0.772 | 0.79 | No |
| 344 | POTCHA | 5 | 147-161 | 0.666 | 0.743 | No |
| 345 | RM011 | 3 | 98-102 | 0.25 | 0.651 | Yes |
| 346 | RM066 | 3 | 97-113 | 0.142 | 0.423 | Yes |
| 347 | RM094 | 2 | 124-144 | 0.13 | 0.124 | No |
| 348 | RM103 | 7 | 128-144 | 0.772 | 0.788 | No |
| 349 | RM137 | 4 | 151-161 | 0.666 | 0.723 | No |
| 350 | RM153 | 3 | 122-126 | 0.315 | 0.325 | No |
| 351 | RM178 | 5 | 136-148 | 0.695 | 0.715 | No |
| 352 | RM188 | 5 | 121-135 | 0.8 | 0.774 | No |
| 353 | RM192 | 3 | 152-158 | 0.521 | 0.428 | No |
| 354 | RM309 | 2 | 105-107 | 0.000* | 0.466 | Yes |
| 355 | RM321 | 2 | 117-121 | 0.052 | 0.052 | No |
| 356 | RM372 | 6 | 123-141 | 0.809 | 0.813 | No |
| 357 | RM388 | 7 | 133-155 | 0.444 | 0.396 | No |
| 358 | RM6 | 6 | 122-140 | 0.7 | 0.735 | No |
| 359 | RME010 | 5 | 102-110 | 0.652 | 0.672 | No |
| 360 | RME030 | 7 | 234-260 | 0.642 | 0.73 | No |
| 361 | TGLA023 | 4 | 97-103 | 0.681 | 0.626 | No |
| 362 | TGLA159 | 7 | 227-241 | 0.727 | 0.785 | No |
| 363 | TGLA179 | 4 | 115-121 | 0.608 | 0.688 | No |
| 364 | TGLA227 | 5 | 78-86 | 0.772 | 0.738 | No |
| 365 | TGLA231 | 5 | 117-125 | 0.863 | 0.75 | No |
| 366 | TGLA261 | 4 | 249-265 | 0.454 | 0.605 | No |
| 367 | TGLA272 | 3 | 112-116 | 0.391 | 0.503 | No |
| 368 | TGLA28 | 4 | 151-175 | 0.434 | 0.547 | No |
| 369 | TGLA345 | 8 | 108-134 | 0.466* | 0.836 | Yes |
| 370 | TGLA36 | 7 | 122-136 | 0.727 | 0.772 | No |
| 371 | TGLA433 | 4 | 200-206 | 0.818 | 0.756 | No |
| 372 | TGLA436 | 2 | 202-204 | 0.263 | 0.308 | No |
| 373 | TGLA44 | 2 | 148-150 | 0.533 | 0.496 | No |
| 374 | TGLA73 | 4 | 131-141 | 0.416 | 0.628 | Yes |
| 375 | TGLA75 | 9 | 149-179 | 0.791 | 0.849 | No |
| 376 | UMBTL | 2 | 164-174 | 0.434 | 0.486 | No |
| 377 | UMBTL070 | 5 | 156-176 | 0.826 | 0.703 | No |
| 378 | UMBTL187 | 5 | 219-239 | 0.826 | 0.703 | No |
| 379 | UMBTL65 | 4 | 119-131 | 0.434 | 0.599 | No |
| 380 | URB002 | 5 | 123-139 | 0.375 | 0.552 | No |
| 381 | URB014 | 6 | 117-137 | 0.526 | 0.624 | No |
| 382 | URB028 | 6 | 153-165 | 0.708 | 0.646 | No |
| 383 | URB048 | 8 | 156-182 | 0.666 | 0.797 | No |
| 384 | URB062 | 3 | 223-227 | 0.583 | 0.52 | No |
| 385 | URB067 | 2 | 113-131 | 1.000* | 0.515 | No |
| 386 | URB068 | 6 | 113-133 | 0.444 | 0.593 | No |
| 387 | UW29 | 6 | 78-92 | 0.666 | 0.726 | No |
| 388 | UWCA20 | 7 | 89-111 | 0.761 | 0.68 | No |
| 389 | UWCA25 | 6 | 113-125 | 0.681 | 0.664 | No |
| 390 | X67827 | 3 | 97-101 | 0.409 | 0.519 | No |
| 391 | Z27076 | 6 | 166-176 | 0.619 | 0.659 | No |

* Loci are significantly (P <0.05) deviated from Hardy-Weinberg equilibrium
