# Supplementary material for: Microsatellite markers of water buffalo, Bubalus bubalis - development, characterisation and linkage disequilibrium studies
Source: BMC Genet. 2009 Oct 21;10:68. doi: 10.1186/1471-2156-10-68 (PMC2773805; doi:10.1186/1471-2156-10-68)
Supplement: Additional file 2 — Monomorphic microsatellite loci of Bubalus bubalis developed through cross species amplification. All these markers were originally developed for cattle. [file 1471-2156-10-68-S2.DOC]

**Monomorphic microsatellite loci of *Bubalus bubalis* developed through cross species amplification. All these markers were originally developed for cattle.**

| S.No | Locus |
| --- | --- |
| 1 | AE129 |
| 2 | AGLA17 |
| 3 | AGLA227 |
| 4 | AGLA293 |
| 5 | AR023 |
| 6 | BB1543 |
| 7 | BL1080 |
| 8 | BL5 |
| 9 | BL50 |
| 10 | BM103 |
| 11 | BM1545 |
| 12 | BM1547 |
| 13 | BM1856 |
| 14 | BM1857 |
| 15 | BM3026 |
| 16 | BM3627 |
| 17 | BM6506 |
| 18 | BM6526 |
| 19 | BM7225 |
| 20 | BM733 |
| 21 | BM764 |
| 22 | BM8125 |
| 23 | BMS1207 |
| 24 | BMS1224 |
| 25 | BMS1237 |
| 26 | BMS1247 |
| 27 | BMS1300 |
| 28 | BMS1510 |
| 29 | BMS1864 |
| 30 | BMS1866 |
| 31 | BMS1898 |
| 32 | BMS1907 |
| 33 | BMS2076 |
| 34 | BMS2168 |
| 35 | BMS2315 |
| 36 | BMS2569 |
| 37 | BMS362 |
| 38 | BMS419 |
| 39 | BMS468 |
| 40 | BMS482 |
| 41 | BMS501 |
| 42 | BMS529 |
| 43 | BMS650 |
| 44 | BMS6506 |
| 45 | BMS764 |
| 46 | BMS803 |
| 47 | BMS836 |
| 48 | HEL5 |
| 49 | IDVGA62A |
| 50 | IDVGA90 |
| 51 | INRA027 |
| 52 | INRA088 |
| 53 | INRA123 |
| 54 | INRA129 |
| 55 | INRA145 |
| 56 | MAF070 |
| 57 | MB009 |
| 58 | RM012 |
| 59 | RM032 |
| 60 | TEXAN2 |
| 61 | TGLA116 |
| 62 | TGLA164 |
| 63 | TGLA226 |
| 64 | TGLA245 |
| 65 | UMBTL184 |
| 66 | URB037 |
