# Supplementary material for: Microsatellite markers of water buffalo, Bubalus bubalis - development, characterisation and linkage disequilibrium studies
Source: BMC Genet. 2009 Oct 21;10:68. doi: 10.1186/1471-2156-10-68 (PMC2773805; doi:10.1186/1471-2156-10-68)
Supplement: Additional file 3 — Characteristics of polymorphic microsatellite loci developed from an enriched genomic library of Bubalus bubalis. [file 1471-2156-10-68-S3.DOC]

**Characteristics of polymorphic microsatellite loci developed from an enriched genomic library of *Bubalus bubalis*.**

| S.No | Locus | Primer sequence | | Ann.  Tem. (^o^C) | No.of Alleles | Allele size | He. obs | He. exp | Null Allele Presence |
| --- | --- | --- | --- | --- | --- | --- | --- | --- | --- |
|  |  | Forward (5’-3’) | Reverse (5’-3’) |  |  |  |  |  |  |
| 1 | **CCMB001** | CCTCCCAACACACAACACAC | CAGCTCCATTCCCATTTCAC | 52 | 13 | 200-224 | 0.833 | 0.887 | No |
| 2 | CCMB005 | AAGTAACGGAGCACACCAGAGT | AAGTGAGACTGGATTCCGACAG | 60 | 17 | 178-202 | 0.909 | 0.929 | No |
| 3 | CCMB006 | TGAGGAAGAAGCCAGGTGAG | TTCCGAGGAATTCAGTCCAG | 52 | 9 | 113-133 | 0.667 | 0.668 | No |
| 4 | CCMB009 | ATGGTGTGGGCAGAGAGAGAC | CCATCCATGCACGTTATCCT | 58 | 12 | 147-177 | 1 | 0.732 | No |
| 5 | CCMB010 | GCGCTACATGTCTATGGTTGTG | GTTTAAGTGGCTGCTTCAGAGG | 50 | 2 | 118-120 | 0.083 | 0.082 | No |
| 6 | CCMB011 | CCCCCAGAACATTTCAGAGC | ACATGGATGTCCCAGAAGACAG | 52 | 7 | 114-124 | 0.625 | 0.762 | No |
| 7 | CCMB014 | TGATATGCAGTATCCTATTGGTTG | GGAAGCTTGGTGAAAAGCAA | 52 | 6 | 155-163 | 0.565 | 0.72 | No |
| 8 | CCMB016 | GGGATAGGAAGTCTCAGGTGAA | CGGAGAATGGTCTTTGGCTA | 52 | 9 | 181-201 | 0.542 | 0.721 | No |
| 9 | CCMB019 | TCTGTTCGGGGAACTGAGAT | CCTCCTAAGGACAAAGCTGGTA | 52 | 6 | 164-173 | 0.444 | 0.657 | No |
| 10 | CCMB023 | TTCTGCTCTAGGCTCAACTGTG | AAACCCAAGCAGTCTGGCTTAC | 50 | 10 | 177-194 | 0.542* | 0.85 | Yes |
| 11 | CCMB026 | GCAGAGAGAGACCCATCCAG | CCCTCGAGGTCGACGGTATC | 52 | 2 | 195-205 | 1.000* | 0.511 | No |
| 12 | CCMB027 | CCCATGGGGAAGAAAATTCC | ACTCAGGCACAGTGTTTGTCAC | 50 | 7 | 149-159 | 0.792 | 0.775 | No |
| 13 | CCMB029 | CTGTGTCCATCACACACACCAT | GTCAGCCCAGACCACCTAAG | 52 | 8 | 144-172 | 0.667 | 0.749 | No |
| 14 | CCMB031 | GGGTCACAAAGGGTCAGACAAA | CACTCCTCCTGAATTCCTGTCT | 52 | 6 | 100-114 | 0.917* | 0.761 | No |
| 15 | CCMB042 | AGATACACACACCGCTCATCAC | CAAGCACCTGTTCATACCTCTG | 58 | 11 | 154-184 | 0.889 | 0.773 | No |
| 16 | **CCMB045** | CTCTCACTGCAGCTCCATCC | TTTACCTCTGACACCTCCCAAC | 52 | 14 | 229-255 | 0.833 | 0.908 | No |
| 17 | CCMB046 | CGTTAGTGATGCCTGTGACTTC | GTGCTGTTGTGAGTTTGAGCTG | 52 | 7 | 140-158 | 0.917* | 0.754 | No |
| 18 | CCMB048 | GAATGGATGCAGGACAGATG | AGAGAGTCAGCTGCGACTAGGT | 62 | 10 | 114-138 | 1 | 0.845 | No |
| 19 | CCMB054 | CCTTGCACTCAGACAGACAGAC | AGCTGTTCAGGTAGGAATCAGG | 52 | 8 | 151-175 | 0.6 | 0.756 | No |
| 20 | CCMB056 | GGCAACCTGAAGGTCAACTTAT | GAACTGACAGGGGCAGTGTGT | 52 | 5 | 127-132 | 0.696 | 0.675 | No |
| 21 | CCMB057 | GTCGCAAAGAGTCAGACACGAC | GATACCTATCTCACATGGCTCGT | 52 | 4 | 108-112 | 0.500* | 0.605 | No |
| 22 | CCMB058 | AAACAGGGATGGACTTTCAAGA | GGGCTATACAGTCCATGAGGTC | 52 | 4 | 128-134 | 0.476 | 0.443 | No |
| 23 | **CCMB059** | ATACTCCCCGTAGATCAGGAG | CTCTGTGTCTGTGTGTGTGTGC | 52 | 4 | 101-109 | 0.35 | 0.31 | No |
| 24 | **CCMB060** | CCACTTGGTTTCAAGGCTCAG | CACATACACACCCCTCCTATCA | 56 | 6 | 274-281 | 0.75 | 0.657 | No |
| 25 | CCMB062 | GGGGCATTCAGTAAAGAACACT | AACACACACACACAGTGACCAA | 52 | 8 | 113-139 | 0.652 | 0.638 | No |
| 26 | CCMB063 | TCCCTAAGCATAACTTCATTAGCC | CAGGCATCTACCTGACACAGAG | 50 | 12 | 159-180 | 0.875 | 0.862 | No |
| 27 | CCMB065 | CAGGAATTCATACGGGAACTGT | CTGCAGAAGCCTAATGGAGTG | 52 | 6 | 172-182 | 0.667 | 0.724 | No |
| 28 | CCMB066 | CCTAAGCATAACTTCATTAGCAAGC | AGGGCATCTACCTGACACAGAG | 50 | 11 | 158-179 | 0.917 | 0.89 | No |
| 29 | CCMB071 | TCAAACCTGCATTTCTTGTGTC | CCTTTCACAGAATCCCCCTTA | 50 | 12 | 224-247 | 0.875 | 0.885 | No |
| 30 | CCMB074 | GCAGCTTTTGGAGTTCCTTCT | CCATGAAGCCTTAGAATCAGGT | 50 | 9 | 113-138 | 0.652 | 0.604 | No |
| 31 | CCMB077 | AGTCAACGCTCGCTGAGAAG | ACGAGAAGGTATGGGTGTGTCT | 52 | 7 | 234-250 | 0.87 | 0.752 | No |
| 32 | CCMB078 | TTTGAAGGTAAACTGGCAGGAT | CAATCTGATTCACTTGGGATGA | 50 | 7 | 124-138 | 0.625 | 0.649 | No |
| 33 | CCMB082 | ACTTCGTGTGTGTGTCTCCATC | ACACCCAGTCCCTTTGTGTAAC | 58 | 7 | 182-203 | 0.684 | 0.826 | No |
| 34 | CCMB088 | CAGAGATGTCGAAATGTTGTCC | CTCTAAAGTGGCAGAGCCTCA | 52 | 5 | 137-149 | 0.545 | 0.517 | No |
| 35 | CCMB090 | GGTCAGGGCTGTGTCTCTGT | CGGAGTCCTCTGGTTCACAT | 52 | 4 | 164-173 | 0.350* | 0.642 | Yes |
| 36 | CCMB091 | GTGGGTGGGTGAAGTGAGTG | ACACCCTCACACACACTTATATT | 50 | 2 | 127-138 | 0.042 | 0.049 | No |
| 37 | CCMB093 | CTGACGACACATTCACTCCAAT | CCGTTCTCGTTTTTAAGCAATA | 52 | 10 | 306-320 | 0.875 | 0.829 | No |
| 38 | CCMB096 | AAATTGGAATGGGTGGAGTGT | CACCACAAACAACATACACACG | 52 | 9 | 282-308 | 0.583 | 0.78 | Yes |
| 39 | CCMB099 | CCTCCTCTTCTGATTGCTTGAC | AATCACGTTTGTTGCCTGTTTT | 52 | 9 | 113-133 | 0.625 | 0.646 | No |
| 40 | CCMB105 | AGGAGAGAGACGTCAAGAGTGG | ACAGCTTCGCCTCTTTTGTG | 52 | 12 | 166-194 | 0.833 | 0.859 | No |
| 41 | CCMB107 | CTGAAGCCACAGAACCAAATG | TCAGAAATCTGCTTCCTTATTTCA | 52 | 9 | 169-185 | 0.667 | 0.74 | No |
| 42 | CCMB108 | GTCATAGATCAGCACCCTACGC | CGTGTCTCTGCCATTAGACGTA | 52 | 7 | 107-119 | 0.682 | 0.727 | No |
| 43 | CCMB109 | TTGTTGGGGAAGGATTCAAG | CGACACATTCACTCCAATCG | 52 | 4 | 97-105 | 0.292 | 0.269 | No |
| 44 | CCMB112 | GAATGTCAGGCACGGATG | TTCGTGTGTGTGTCTCCATCT | 52 | 7 | 116-134 | 0.826 | 0.792 | No |
| 45 | CCMB113 | TTTTCCTATCTGCCCCACCT | TACCACTGTGAAGCGATGGA | 58 | 11 | 197-218 | 0.826 | 0.873 | No |
| 46 | CCMB114 | GAAACAGGCCAGGGGACT | ATGGGTGACTGAGCACATGA | 52 | 7 | 137-153 | 0.87 | 0.764 | No |
| 47 | **CCMB115** | TCTAAGACCACCACCCACCT | GAGAGGGAGAGAGGGGAAGA | 50 | 4 | 202-215 | 1.000* | 0.588 | No |
| 48 | CCMB116 | CGTATCGAGCCTGTGTGTGT | GTGCCTCCTCTTCTGATTGC | 60 | 11 | 143-181 | 0.682 | 0.783 | No |
| 49 | CCMB117 | GCTTTGGGAAGAATGCAGAG | GCAGCTTTTGGAGTTCCTTC | 60 | 12 | 154-196 | 0.625 | 0.694 | No |
| 50 | CCMB118 | TTTGAAGGTAAACTGGCAGGA | TTAGGAAATATCATGAACTATCTGCAT | 60 | 12 | 148-170 | 0.917 | 0.856 | No |
| 51 | CCMB121 | ATCCGCCACAGGTTCATATT | GTTTCTTGCCTGGACAATCC | 60 | 6 | 189-196 | 0.375 | 0.54 | Yes |
| 52 | CCMB122 | ACCCTCTTCTTCCCTCTCCA | TTGCAGGCACAGAAACTGAC | 60 | 10 | 116-196 | 0.875 | 0.67 | No |
| 53 | CCMB123 | GGAAACCAGGTCCTGAGTGA | GCTTTGGAGAATTTCCATTATTTT | 60 | 9 | 186-199 | 0.500* | 0.826 | Yes |
| 54 | CCMB124 | GTCCCCATAGGACCAAAACC | AATGTGAAGCAGAGCCCAAC | 60 | 10 | 177-195 | 0.762 | 0.877 | No |
| 55 | CCMB125 | GGAAAAACCATAGCTTTGACTAGA | GCCTGGTAGGCTGCCATCT | 60 | 11 | 140-156 | 1.000* | 0.803 | No |
| 56 | CCMB126 | CCAAGGATAGAAGAGCCTGGT | CCCGGAAGACTGACAGTTGA | 60 | 9 | 136-157 | 0.917 | 0.851 | No |
| 57 | CCMB127 | TTCACCTGTTTTATTGTTTTTAAATGA | CATCCATTGGGTTCACACAG | 60 | 19 | 148-180 | 0.458 | 0.686 | Yes |
| 58 | CCMB128 | CTGGTGGGCTGCCATCTC | TGATACAGCAGTGGCACAGA | 58 | 3 | 197-217 | 1.000* | 0.539 | No |
| 59 | CCMB129 | GGGACACATATGTAATGCCTCA | GGTCCTAGCTACTTCCATCTAGCA | 60 | 6 | 146-164 | 0.958* | 0.738 | No |
| 60 | CCMB130 | TGGTGGGCTGCCATCTAT | GATGAGGAAACTAGGACTCAGGA | 60 | 9 | 145-157 | 0.5 | 0.638 | No |
| 61 | CCMB131 | TTCACAAGTGCTTTCATTAACAGT | ATCCCAGGGACGGAGGAG | 60 | 8 | 184-197 | 0.250* | 0.406 | Yes |
| 62 | CCMB132 | TCCTCCTCCTTAAAGGCACT | TCTAAGAAGCAAAGCTCTGACG | 60 | 5 | 170-175 | 0.455 | 0.706 | No |
| 63 | CCMB133 | CACTAGTAAACGTGAGTAAAGAAATGC | CAAATTTTTACTTTTTATGGCTGAG | 60 | 11 | 166-185 | 0.182* | 0.834 | Yes |
| 64 | CCMB134 | GCTGTTCACTGATTGAAACATACC | GCCCTACCCACAACACACAT | 60 | 7 | 162-180 | 0.25 | 0.306 | No |
| 65 | CCMB135 | ATCCCAGAGAGAAAAGCACTACAG | TGAGGTTTTCTTTGGGTTTCC | 60 | 16 | 147-180 | 0.75 | 0.917 | Yes |
| 66 | CCMB136 | TGTTCTTGCCTGGAGAATCC | CATGAAACCAAAAATGTAAACCA | 60 | 3 | 192-195 | 0.211 | 0.482 | Yes |
| 67 | CCMB137 | GAATCCCAGGGACAGAGGAG | CAAGAACGTGTCACCTAAACCA | 60 | 6 | 162-170 | 0.125* | 0.734 | Yes |
| 68 | CCMB140 | GGGGGTAAACAAGTCCCAGT | CAGCTCTCCTTCTCAACTTTCC | 60 | 6 | 177-183 | 0.130* | 0.415 | Yes |
| 69 | CCMB141 | CCAACTTCATCAGCAACAGG | ATTAGTGTATTACTCTGGGTTCTGC | 50 | 6 | 140-152 | 0.636 | 0.814 | No |
| 70 | CCMB142 | GCAAATTGCTGAGTGGATCA | TGCACTCAAAAGAAGTGAGGAA | 60 | 9 | 163-190 | 0.792* | 0.848 | No |
| 71 | CCMB144 | CTGTTGGCCATATGAATTTCC | TGCAAATGATATGACTGGTAAAGAA | 60 | 6 | 162-169 | 0.208* | 0.603 | Yes |
| 72 | CCMB146 | TCTCAGGAAACCTCTTTCTCCA | CGGGCTAACTGTCCATGC | 60 | 9 | 150-167 | 0.458* | 0.705 | Yes |
| 73 | CCMB148 | TATGGCTGGGGGTTAGATTG | GTGATGTGGGGAGAGGAGAG | 54 | 4 | 184-190 | 0.273 | 0.326 | No |
| 74 | CCMB149 | AGGAGAAGTGGGGCTTCTGT | GGGAACTCTTGGCTATCCATC | 60 | 6 | 168-193 | 0.250* | 0.431 | Yes |
| 75 | CCMB152 | TTTCTGTACCTTTTGCTGAATTTG | GGCTCTGTTTGCAGATGACA | 58 | 5 | 110-122 | 0.7 | 0.592 | No |
| 76 | CCMB153 | GTTACAGTCCATGGGGTTG | TTGGTTTTAAATGCAATATAGG | 60 | 9 | 173-184 | 0.619* | 0.878 | Yes |
| 77 | CCMB154 | TTGCTATATCATGCTTGTCTACTGA | GGTCACACACAGTCGGACAC | 60 | 8 | 114-130 | 0.652 | 0.65 | No |
| 78 | CCMB155 | TTTCAAATTGCCCATTTATCC | TGCTGTGTGACTAACCTTCTGAG | 60 | 4 | 113-116 | 0.375* | 0.754 | Yes |
| 79 | CCMB159 | GAGCCCTCCTGACTCCAGA | ACCAAGATGCTGACATGTGG | 60 | 11 | 159-175 | 0.75 | 0.868 | No |
| 80 | **CCMB164** | AGTTCTTGGGTGACCAGGTG | AACTTTCTGCCTGTGCTCTTT | 60 | 8 | 185-201 | 0.696 | 0.731 | No |
| 81 | CCMB168 | GGCACAGCACATGCTTAGAA | ACCAGGCCAGGTATTCTTCC | 60 | 7 | 101-116 | 0.783 | 0.796 | No |
| 82 | CCMB169 | AGGACAACTAAGGGGTGTGG | TTGCCCTTATTTGCTTCTCTTC | 54 | 8 | 144-170 | 0.739 | 0.851 | No |
| 83 | CCMB170 | TTTGTGTTACATTTACGTAGCAAG | CCAGAATCTTACTCTAAACAAGCTG | 60 | 9 | 150-178 | 0.958* | 0.779 | No |
| 84 | CCMB175 | TCCCACTGCTTTCTTTGTGTT | GATGTAATGGAGGCTTTAGGG | 60 | 8 | 154-176 | 0.875 | 0.723 | No |
| 85 | CCMB179 | TCAGAAAAGCAAAGGGCTGT | TTTCTACAAACCAGATCTATCAGCA | 60 | 10 | 105-132 | 0.417* | 0.683 | Yes |
| 86 | CCMB181 | CCTGCAAGCTAAACACATGTACC | TGGCTTCTTCAACTGACCCTA | 60 | 7 | 160-174 | 0.348* | 0.557 | Yes |
| 87 | CCMB184 | TCTGTCTACGCTCACCTCCA | GTTTCTCTCCATCGCAGCAT | 56 | 2 | 184-186 | 0.143 | 0.136 | No |
| 88 | CCMB190 | GGCATCCTTGGATTCAGTTG | GAAAGGCAGAGGGGAGATTC | 60 | 8 | 168-184 | 0.375* | 0.543 | Yes |
| 89 | CCMB198 | GCAAATCAACAATTGTGACCCTA | TAACAGAGGCTGGAGGGAAA | 60 | 9 | 163-183 | 0.875 | 0.777 | No |
| 90 | CCMB199 | TCATTGCAAATTTTGTATCTATTGG | TCCATGTGACAGAAGAGCAGA | 60 | 10 | 162-186 | 0.875 | 0.865 | No |
| 91 | CCMB201 | GACAGGACATGCACAGCTTC | GACCAGCTGCTTTCTTCAGC | 60 | 10 | 146-170 | 0.739 | 0.849 | No |
| 92 | CCMB202 | TCTCTTGCTTGAATTCGGACT | CCTGGAGGAGGGAAAAGCTA | 60 | 14 | 150-167 | 0.609* | 0.848 | Yes |
| 93 | CCMB204 | CCATTGGTCCTAGAGGCTGA | GGATCCGATTCTCTTGCTTG | 60 | 4 | 165-175 | 0.000* | 0.6 | Yes |
| 94 | CCMB206 | AAATTCCATGGACAGAGGAG | TGGTTAGAACACAGGCTCTGAA | 60 | 12 | 150-163 | 0.708* | 0.795 | No |
| 95 | CCMB207 | CTGACGTGTTTTCTGTTGAATAGG | GAACAGTCCTCTCACAAAACAGTG | 60 | 13 | 150-174 | 0.958 | 0.907 | No |
| 96 | CCMB208 | GAAGCGAGTTAGTGCACACG | CAGCAGTCCCTGTTGAGACA | 60 | 5 | 246-252 | 0.556 | 0.633 | No |
| 97 | CCMB211 | AAAGGCACATTTTCTCCCTTC | CAGCCCAAGAACCTAGGAGA | 60 | 12 | 152-178 | 0.875 | 0.818 | No |
| 98 | CCMB213 | AGACCTGGCCAAGACCTGT | GGGGCTTGGGGAGTGAAG | 60 | 10 | 162-184 | 0.826 | 0.826 | No |
| 99 | CCMB215 | CCATGTGGCCAGCATTAGTA | TCACCTAGAAAAATGAGATTGTG | 60 | 8 | 157-173 | 0.792 | 0.77 | No |
| 100 | CCMB221 | GAAATAAGAACTATTTCCAGGTAGC | TAGAGTGCACATGTGTCTGTGTG | 58 | 6 | 142-152 | 0.864* | 0.762 | No |
| 101 | CCMB223 | TTGCCTTTTCATCTGTGTTTG | TGACAGCTGCGTACTGAAGG | 60 | 4 | 131-137 | 0.083 | 0.122 | No |
| 102 | CCMB225 | ACATCTAACACCTCACATAGTCTG | CAATGAACAGCTTCGTGACTACA | 60 | 5 | 153-158 | 0.042* | 0.551 | Yes |
| 103 | CCMB229 | CCATAGGAAGATGTGTGACACTG | CCTGAATTCCTAGTTTTGTTGTG | 60 | 17 | 150-168 | 0.609* | 0.935 | Yes |
| 104 | CCMB236 | AGGGGTGTGTAACACCAGGA | CGCTCAAAGTCACACAGACA | 58 | 2 | 162-164 | 0.15 | 0.224 | No |
| 105 | CCMB237 | CAGGTGGGTTCGTTACCACT | TCAAACAAGTGGAATCAAAATGTC | 58 | 5 | 197-203 | 0.5 | 0.651 | No |
| 106 | CCMB238 | TCTTCTATACAGCAAAGTGATGAGC | TGTGATAAACCACAGTGGAAAA | 60 | 10 | 90-108 | 0.833 | 0.814 | No |
| 107 | CCMB239 | ATTCTCCTCTCCAGGGCATC | TCATTTTAGCAGCGATGCAG | 60 | 6 | 146-160 | 0.391* | 0.642 | Yes |

*Loci are significantly (P <0.05) deviated from Hardy-Weinberg equilibrium; Loci amplified on Chinese hamster cell line are in bold.
