# Supplementary material for: Microsatellite markers of water buffalo, Bubalus bubalis - development, characterisation and linkage disequilibrium studies
Source: BMC Genet. 2009 Oct 21;10:68. doi: 10.1186/1471-2156-10-68 (PMC2773805; doi:10.1186/1471-2156-10-68)
Supplement: Additional file 4 — Monomorphic microsatellite loci derived from an enriched genomic library of Bubalus bubalis. [file 1471-2156-10-68-S4.DOC]

**Monomorphic microsatellite markers derived from an enriched genomic library of *Bubalus bubalis***

| S.No | Locus | Primer sequence | | | Annealing  Temp. (^o^C) |  |
| --- | --- | --- | --- | --- | --- | --- |
|  |  | Forward (5’-3’) | Reverse (5’-3’) | |  |  |
| 1 | CCMB052 | TCACTATAGGGCGAATTGGAG | | TCACACAACCACAGCACTCA | 52 |  |
| 2 | CCMB092 | TTGATAGGCTTTTGTGATGAGC | | CCAGGTGCTAGAGCAGAGACTA | 52 |  |
| 3 | CCMB098 | AAATTGGAATGGGTGGAGTGT | | AGCTCAGACCAGATGCTTTTTC | 52 |  |
| 4 | **CCMB139** | CTATGGGGTCGCAGAGTCA | | CTGATGTGTTTCTCTCTGGCTTT | 50 |  |
| 5 | CCMB158 | TGAACATCTGAGTGAAAGTCACAG | | TCTGCCCAGAGATCACCATT | 60 |  |
| 6 | CCMB176 | CATAATCAGTCACATACCCTTTACC | | TCAAAATATCTATCAAAAGGGTTAG | 60 |  |
| 7 | CCMB177 | GGAGTGGCCTCTAGAGATTCTG | | CAGATGCATAGGGAACCTCAG | 60 |  |

Locus amplified on Chinese hamster cell line is in bold
